# Molecular data and ecological niche modeling reveal population dynamics of widespread shrub *Forsythia suspensa* (Oleaceae) in China’s warm-temperate zone in response to climate change during the Pleistocene

**DOI:** 10.1186/1471-2148-14-114

**Published:** 2014-05-30

**Authors:** Zi-Zhen Fu, Yong-Hua Li, Kai-Ming Zhang, Yong Li

**Affiliations:** 1 College of Forestry, Henan Agricultural University, Zhengzhou 450002, China

**Keywords:** cpDNA, nrDNA, Ecological niche model, China’s warm-temperate zone, Molecular phylogeography, *Forsythia suspensa*

## Abstract

**Background:**

Despite its high number of endemic deciduous broad-leaved species in China’s warm-temperate zone, far less attention has been paid to phylogeographic studies in this region. In this work, the phylogeographic history of *Forsythia suspensa* endemic to China’s warm-temperate zone was investigated to explore the effect of climate change during the Pleistocene on the distribution of this deciduous broad-leaved species in China.

**Results:**

The cpDNA data revealed seven phylogeographical groups corresponding to geographical regions. By contrast, the nrDNA data supported the samples clustered into three groups, which was inconsistent with separate geographical regions supported by cpDNA data. Ecological niche modeling showed that the climatically suitable area during the cold period was larger than that during the warm period.

**Conclusions:**

Both molecular data and ecological niche modeling indicated that *F. suspensa* expanded to nearby low-elevation plains in the glacial periods, and retreated to mountaintops during interglacial warmer stages. This study thus supported that *F. suspensa* persisted *in situ* during the glacial of the Pleistocene with enlarged distribution area, contrary to the hypothesis of long distance southward migration or large-scale range contraction.

## Background

Profound climatic oscillations during the Pleistocene resulted in repeated drastic environmental changes, which also substantially shaped the species’ distribution, evolution, and extinction [1-4]. The Last Glacial Maximum (LGM) occurred at around 23 000–18 000 years before present, which was particularly considered an important factor influencing the current plant distribution [4,5]. Numerous molecular-based phylogeographical surveys in Europe and North America have been extensively studied to unravel the locations of refugia, the potential recolonization routes and the subsequent evolution and speciation during glacial and post-glacial epochs [6-8]. In contrast to Europe and North America, most parts of China were not covered by massive ice sheet during LGM. It was clear that ice sheet had only developed in certain areas of the Qinghai–Tibetan Plateau [9]. In recent years, numerous phylogeographical surveys of plant species in China have been informative in resolving the location of glacial refugia and routes of colonization/range expansion after glacial periods.

Three hypotheses on the occurrence of refugia and postglacial expansion in China have been proposed. The first hypothesis is that climatic changes during Pleistocene deeply impacted the forests in China, and large-scale vegetation experienced long-distance southward migration during glaciations. Thus, the species subsequently colonized northward from the southern refugia after glaciation [10-12]. The second hypothesis is that climatic changes during Pleistocene had a large influence on the forests in China; however, no large-scale vegetation experienced long-distance southward migration during glaciation and instead contracted into a few main refugia, as suggested by most phylogeographical studies in China [13,14]. The third hypothesis is that some species were slightly affected by Pleistocene glacial cycles that persisted *in situ* throughout the LGM and occupied multiple localized glacial refugia, as suggested by some phylogeographical studies in this region on species such as *Taxus wallichiana*[15], *Cathaya argyrophylla*[16], and *Eurycorymbus cavaleriei*[17]. Some species reportedly occupied a much larger area than today, e.g., *Alsophila spinulosa*[18], *Pinus kwangtungensis*[19], and *Primula obconica*[20]. Nevertheless, these studies have mainly focused on the plant species in the regions with high biodiversity, such as Qinghai–Tibetan Plateau, the Himalaya–Hengduan Mountains, and subtropical China, and only a few studies are about China’s warm-temperate zone. However, this region has the highest population density in the country and has developed agriculture. Dramatic climate change is adversely affecting the global ecological system and agricultural cultivation. Therefore, more comprehensive studies on species with wide distribution ranges in China’s warm-temperate zone are needed to test the impact on this region during the Pleistocene glacial cycle.

“China’s warm-temperate zone” generally refers to the area between 32°30′–42°30′ and 103°30′–124°10′. In this region, the typical vegetation is deciduous broad-leaved forest (DBLF) [21]. Since the Tertiary Period, the warm-temperate zone in China has not been strongly influenced by massive Quaternary glaciers. In contrast to the extinction of a large number of broad-leaved species in Europe and North America, the majority of deciduous broad-leaved species is preserved in this region [22]. Although they have survived the Quaternary glacial period, the evolution and distribution of the warm-temperate DBLF were still affected by climate fluctuations. However, our knowledge on phylogeographical histories of organisms occurring in China’s warm-temperate zone and their correlations with climatic fluctuations has been limited due to finite phylogeographical studies in this region, particularly for plants [10,23-25]. Thus, increased attention must be paid to China’s warm-temperate zone and to the influences of Quaternary climate change in this area based on large-scale genetic studies with extensive sampling.

*Forsythia suspensa* (Thunb.) Vahl (Oleaceae) is a deciduous shrub widely distributed in China’s warm-temperate zone with elevations of 300 m to 2200 m above sea level. As a typical component of DBLF, *F. suspensa* exists in most distribution areas of current DBLF in China. Consequently, we selected *F. suspensa* as a model for inferring phylogeographical patterns in China’s warm-temperate zone to understand DBLF population dynamics in response to climate change.

In this study, two chloroplast DNA (cpDNA) regions, one nuclear ribosomal DNA (nrDNA) region, and ecological niche models were used to examine the phylogeographical pattern of *F. suspensa*. Our specific objectives were to address the following questions: (i) what is the genetic structure of *F. suspensa* populations in China as revealed by cpDNA and nrDNA data; and (ii) how did the species response to the climatic oscillations during the Pleistocene.

## Results

### cpDNA diversity and population structure

**Figure 1:**
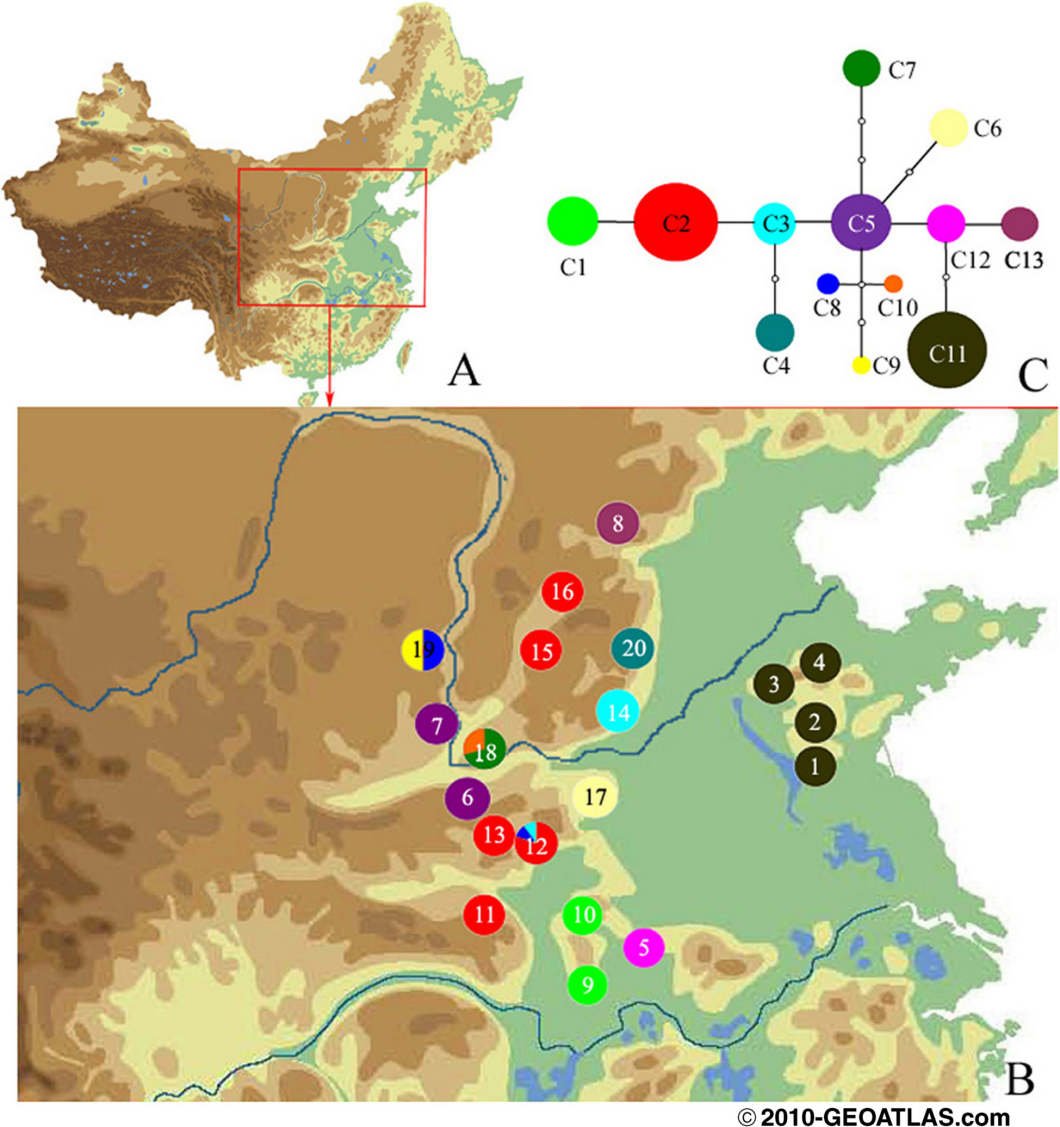
**Geographic distribution and genealogical relationships of cpDNA haplotypes recovered from *F. suspensa *populations in China.** Map was downloaded from http://www.geoatlas.com. **A**, Distribution ranges of *F. suspensa* (red lines) in China. **B**, The geographic distribution of thirteen chlorotypes (C1–C13) detected (for population numbers see Table 1). **C**, The statistical parsimony network of chlorotypes C1–C13. The area of circles corresponds to the frequency of each haplotype. Each solid line represents one mutational step interconnecting two haplotypes for which parsimony is supported at the 95% level.

Out of the two cpDNA regions sequenced in *F. suspensa* (182 individuals, 20 populations), the two regions both showed length variation (*psb*A-*trn*H, 370 bp to 397 bp; *trn*L-F, 782 bp to 783 bp). When combined, these sequences (1153 bp to 1180 bp) were aligned with a consensus length of 1180 bp, and contained 16 nucleotide substitutions and 3 indels. Based on these polymorphisms, 13 chlorotypes (C1 to C13) were identified among all samples surveyed (Additional file 1). The 11 *psbA*-*trnH* and 7 *trnL*-*F* chlorotype sequences were deposited in the GenBank database under accession numbers KF366077 to KF366094. Among the 13 chlorotypes detected, the most widespread chlorotypes were C1 (in 4 of 20 populations) and C2 (in 5 of 20 populations). The geographical distribution of chlorotypes C1 to C13 and their occurrence at each locality are shown in Figure 1B and Table 1. Only 3 of the 20 populations were polymorphic (P12, P18, and P19), whereas the other populations exhibited only one chlorotype. The statistical parsimony network of chlorotypes C1 to C13 revealed that they were only one to seven mutational steps apart (Figure 1C). The haplotype and nucleotide diversities of cpDNA were *h*_T_ = 0.600 and *π*_T_ = 1.58 × 10^−3^, respectively. The highest nucleotide and haplotype diversities were found in P18 and P19, respectively (Table 1). Seven private chlorotypes of populations were found in the species, with the highest in P18.

**Table 1:** **Details of population locations, sample size, cpDNA variation of *F*. *suspensa *** **sampled in China**

**Population no. and code**	**Locations**	**Lat.(N)/ Long.(E)**	***N***	**Chlorotypes (nos. of individuals)**	**π** **×** **10**^**−3**^	***h***	***N*** **pc**
*Shandong group*							
1. SDBD	Baodugu, Shandong	35°00′/117°42′	10	C11 (10)	0	0	0
2. SDMM	Meng Mt., Shandong	35°30′/117°48′	10	C11 (10)	0	0	0
3. SDTM	Tai Mt., Shandong	36°15′/117°06′	10	C11 (10)	0	0	0
4. SDYM	Yuan Mt., Shandong	36°28′/117°51′	10	C11 (10)	0	0	0
*HSS group*							
5. HNJG	Jigong Mt., Henan	31°50′/114°05′	10	C12 (10)	0	0	1
6. SXLJ	Laojun Mt., Shaanxi	34°20′/110°15′	10	C5 (10)	0	0	0
7. SXHM	Hua Mt., Shaanxi	35°33′/110°06′	10	C5 (10)	0	0	0
8. SXWT	Wutai Mt., Shanxi	39°00′/113°35′	8	C13 (8)	0	0	1
*HHS group*							
9. HBDH	Dahong Mt., Hubei	31°31′/112°58′	6	C1 (6)	0	0	0
10. HNTB	Tongbai Mt., Henan	32°23′/112°50′	10	C1 (10)	0	0	0
11. HBWD	Wudang Mt., Hubei	32°24′/111°00′	10	C2 (10)	0	0	0
12. HNLY	Longyuwan, Henan	33°42′/111°45′	10	C2 (8), C3 (1), C8 (1)	0.81	0.378	0
13. HNLJ	Laojieling, Henan	33°45′/111°20′	10	C2 (10)	0	0	0
14. HNJL	Jiulian Mt., Henan	35°35′/113°35′	10	C3 (10)	0	0	0
15. SXLK	Lingkong Mt., Shanxi	36°36′/112°05′	10	C2 (10)	0	0	0
16. SXTL	Tianlong Mt., Shanxi	37°42′/112°26′	6	C2 (6)	0	0	0
*Song Mt. group*							
17. HNSM	Song Mt., Henan	34°28′/113°05′	10	C6 (10)	0	0	1
*Wulaofeng group*							
18. SXWL	Wulaofeng, Shanxi	34°50′/110°35′	10	C7 (7), C10 (3)	1.58	0.467	2
*Baota Mt. group*							
19. SXBT	Baota Mt., Shaanxi	36°35′/109°29′	6	C8 (3), C9 (3)	1.53	0.600	1
*Wuzhi Mt. group*							
20. HBWZ	Wuzhi Mt., Hebei	36°30′/113°39′	10	C4 (10)	0	0	1
Total			186		2.53	0.867	7

π: nucleotide diversity, *h*: haplotype diversity, and *N*pc: number of private chlorotypes.

**Figure 2:**
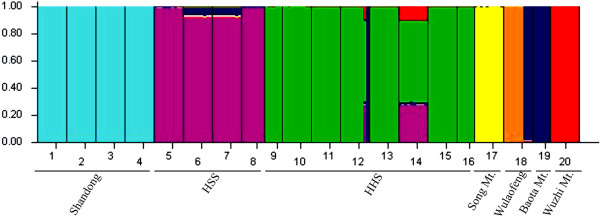
**Estimated genetic structure for *K*** **=** **7 obtained with the program STRUCTURE (Pritchard et al., 2000 **[54]**) for 20 populations of *F. suspensa *based on cpDNA data.** Each vertical bar represents an individual and its assignment proportion (not shown) into one of seven (colored) population clusters (*K*).

Bayesian analysis of population structure (Figure 2) revealed that the highest likelihood of cpDNA data was obtained when samples were clustered into seven groups (*K* = 7; data not shown). The seven groups identified were as follows: Shandong (P1 to P4), Henan–Shaanxi–Shanxi (HSS) (P5 to P8), Henan–Hubei–Shanxi (HHS) (P9 to P16), Song Mt. (P17), Wulaofeng (P18), Baota Mt. (P19), and Wuzhi Mt. (P20) groups. Non-hierarchical AMOVA (Table 2) revealed a strong population genetic structure for cpDNA sequence variation at the species-range scale (*Φ*_ST_ = 0.929; *P* < 0.001). However, most of this differentiation was partitioned among the seven groups (*Φ*_CT_ = 0.793), and only 14.83% was explained among populations within each region (*Φ*_SC_ = 0.715) (Table 2). Significant isolation by geographical distance for cpDNA was detected at the species-range scale (*r* = 0.343; *P* < 0.001). However, the isolation by ecological distance for cpDNA was not significant (*r* = −0.113; *P* > 0.05).

**Table 2:** **Non**-**hierarchical and hierarchical AMOVAs for cpDNA and nrDNA variation surveyed in populations of *F. suspensa *** **in China**

**Source of variation**	**d.f.**	**% Total variance**	***Φ*-statistic**	***P-*** **value**
*cpDNA*				
Non-hierarchical AMOVAs				
Total	19	92.94%	*Φ*_ST_ = 0.929	< 0.001
*Shandong group*	3	NC	NC	NC
*HSS group*	3	100.00%	*Φ*_ST_ = 1.000	< 0.001
*HHS group*	7	83.05%	*Φ*_ST_ = 0.831	< 0.001
Hierarchical AMOVAs				
Among seven groups	6	79.27%	*Φ*_CT_ = 0.793	< 0.001
Among populations	13	14.83%	*Φ*_SC_ = 0.715	< 0.001
Within populations	166	5.90%	*Φ*_ST_ = 0.941	< 0.001
*nrDNA*				
Non-hierarchical AMOVAs				
Total	19	20.66%	*Φ*_ST_ = 0.207	< 0.001
*Shandong group*	3	2.86%	*Φ*_ST_ = 0.029	> 0.05
*HSS group*	3	37.36%	*Φ*_ST_ = 0.374	< 0.001
*HHS group*	7	14.67%	*Φ*_ST_ = 0.147	< 0.001
Hierarchical AMOVAs				
Among seven groups	6	−4.93%	*Φ*_CT_ = −0.049	> 0.05
Among populations	13	24.79%	*Φ*_SC_ = 0.236	< 0.001
Within populations	352	80.15%	*Φ*_ST_ = 0.199	< 0.001

Φ: Fixation index.

**Figure 3:**
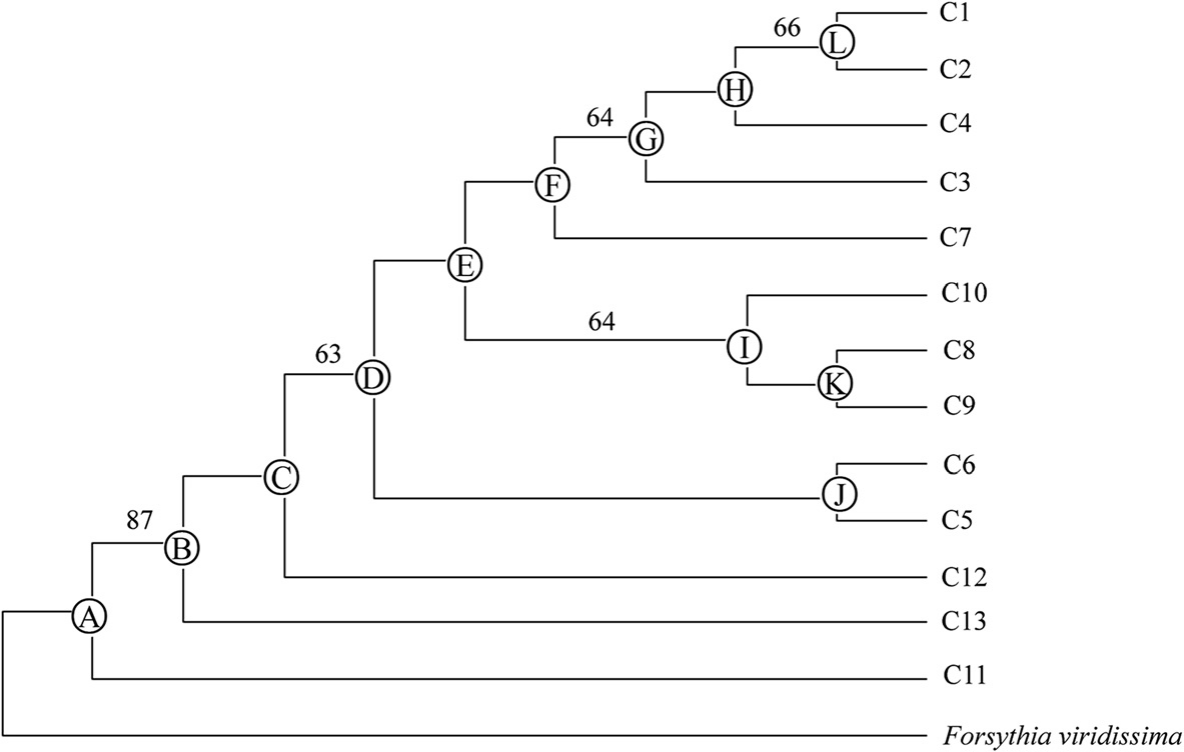
**Neighbor-joining (NJ) clustering of thirteen chlorotype sequences of *F. suspensa. F. viridissima *was used as an outgroup.** The bootstrap confidence values (%) are indicated on the branches.

Tajima’s *D* and Fu’s *Fs* statistics for deviation from neutrality were examined for the four groups including HSS, HHS, Wulaofeng and Baota Mt., and no significant values were detected. The other three groups, including Shandong, Song Mt. and Wuzhi Mt., were not calculated because each of them had a single chlorotype (Table 3). Similarly, mismatch analysis was also not performed for these three groups. Unlike the two neutrality tests, the HSS and Baota Mt. groups showed demographic expansions for nonsignificant SSD and RAG values (Table 3). According to the result of molecular clock calibration, the mean divergence time of nodes ranged from 2.70 Ma (node A) to 0.35 Ma (node L) or 1.90 Ma (node A) to 0.25 Ma (node L) (Figure 3), assuming, respectively, minimum and maximum rates of synonymous substitution in cpDNA [26]. This suggests that the divergence of the species fell into the Early-to-Late Pleistocene.

**Table 3:** **Results of demographic analyses based on two datasets for seven groups and all samples of *F. suspensa***

	**cpDNA**				**nrDNA**			
**Groups**	**SSD (P value)**	**RAG (P value)**	**Tajima’s *D***	**Fu’s *F*** **S**	**SSD (P value)**	**RAG (P value)**	**Tajima’s *D***	**Fu’s *F*** **S**
*Shandong group*	-	-	0	-	0.007 (0.809)	0.026 (0.852)	−1.654*	−14.601**
*HSS group*	0.003 (0.519)	0.082 (0.462)	1.518	1.418	0.022 (0.712)	0.041 (0.822)	−0.772	−3.717
*HHS group*	0.018 (0.041)	0.162 (0.021)	−0.691	0.279	0.327 (0.000)	0.151 (1.000)	−1.769*	−25.668**
*Song Mt. group*	-	-	0	-	0.047 (0.259)	0.123 (0.389)	−0.153	−1.941
*Wulaofeng group*	0.436 (0.000)	0.720 (0.942)	1.229	4.487	0.279 (0.000)	0.466 (0.937)	0.139	0.955
*Baota Mt. group*	0.303 (0.069)	0.880 (0.069)	1.910	2.759	0.077 (0.217)	0.126 (0.433)	−0.665	−0.244
*Wuzhi Mt. group*	-	-	0	-	0.049 (0.020)	0.724 (0.576)	−1.440	−0.674

‘*’, P values, 0.05; ‘**’, P values, 0.01; SSD, sum-of-squared deviations; Rag, Raggedness index.

### nrDNA diversity and population structure

One nrDNA (ITS) region was also sequenced in *F. suspensa* from 186 individuals (20 populations). The aligned sequences were 481 bp and contained 63 nucleotide substitutions. Based on these polymorphisms, 74 ribotypes (R1 to R74) were identified among all samples surveyed (Additional file 2). The sequences of the 74 ribotypes were deposited in the GenBank database under accession numbers KF366003 to KF366076. Among the 74 ribotypes detected, the most widespread haplotype was R2 (in 19 out of 20 populations). The geographical distribution of ribotypes R1 to R74 and their occurrence at each locality are shown in Table 4. Nineteen of the twenty populations were polymorphic, and only P9 exhibited one ribotype. The haplotype and nucleotide diversities based on nrDNA data for *F. suspensa* were *h*_T_ = 0.625 and *π*_T_ = 5.65 × 10^−3^, respectively. Nucleotide diversity (*π*) among the 20 populations ranged from 0 to 23.25 × 10^−3^ and haplotype diversity (*h*) varied between 0 and 0.868. The highest nucleotide diversity and haplotype diversity was found in populations P8 and P3, respectively (Table 4). A total of 61 private ribotypes of populations existed in the species, and the highest number of private ribotype was found in P11.

**Table 4:** **nrDNA variation of *F*. *suspensa *** **sampled in China**

**Population no. and code**	***N***	**Ribotypes (nos. of individuals)**	**π** **×** **10**^**−3**^	***h***	***N*** **pr**
*Shandong group*					
1. SDBD	10	R1 (1), R2 (11), R6 (2), R13 (1), R15 (2), R16 (3)	2.80	0.684	1
2. SDMM	10	R2 (10), R6 (2), R20 (2), R40 (1) R41 (1), R42 (2), R43 (2)	6.47	0.742	3
3. SDTM	10	R2 (7), R16 (2), R26 (1), R40 (1) R47 (1), R48 (1), R49 (3), R50 (1) R51 (1), R52 (1), R53 (1)	8.09	0.868	7
4. SDYM	10	R1 (1), R2 (12), R68 (1), R69 (1), R70 (1), R71 (1), R72 (1), R73 (1) R74 (1)	5.25	0.653	7
*HSS group*					
5. HNJG	10	R1 (6), R2 (14)	0.92	0.442	0
6. SXLJ	10	R1 (1), R2 (9), R29 (1), R30 (1), R31 (3), R32 (2), R33 (1), R34 (1) R35 (1)	3.98	0.789	6
7. SXHM	10	R2 (9), R7 (1), R13 (1), R20 (1), R22 (1), R23 (1), R24 (1), R25 (1) R26 (2), R27 (1), R28 (1)	6.11	0.805	6
8. SXWT	8	R2 (6), R65 (1), R66 (8), R67 (1)	23.25	0.642	3
*HHS group*					
9. HBDH	6	R2 (12)	0	0	0
10. HNTB	10	R1 (2), R2 (14), R3 (1), R4 (1), R5 (1) , R6 (1)	2.05	0.516	2
11. HBWD	10	R2 (10), R37 (1), R54 (1), R55 (1) R56 (1), R57 (1), R58 (1), R59 (1) R60 (2), R61 (1)	7.47	0.758	8
12. HNLY	10	R2 (15), R6 (2), R7 (1), R8 (1), R9 (1)	1.44	0.442	2
13. HNLJ	10	R2 (12), R10 (6), R11 (1), R12 (1)	3.38	0.574	3
14. HNJL	10	R2 (16), R5 (3), R14 (1)	1.32	0.353	1
15. SXLK	10	R2 (16), R36 (1), R37 (1), R38 (1) R39 (1)	2.76	0.368	2
16. SXTL	6	R2 (4), R5 (1), R13 (3), R36 (1), R44 (1), R45 (1), R46 (1)	4.76	0.864	3
*Song Mt. group*					
17. HNSM	10	R2 (15), R5 (4), R13 (1)	1.85	0.416	0
*Wulaofeng group*					
18. SXWL	10	R1 (10), R13 (1), R26 (3) , R34 (1) R37 (2), R62 (1), R63 (1), R64 (1)	4.48	0.742	3
*Baota Mt. group*					
19. SXBT	6	R2 (7), R17 (1), R18 (1), R19 (1) R20 (1), R21 (1)	6.36	0.682	4
*Wuzhi Mt. group*					
20. HBWZ	10	R2 (18), R5 (1), R40 (1)	0.81	0.195	0
Total	186		5.65	0.625	61

π: nucleotide diversity, *h*: haplotype diversity, and *N*pr: number of private ribotypes.

**Figure 4:**
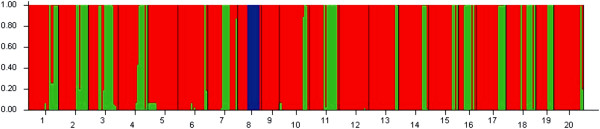
**Estimated genetic structure for *K*** **=** **3 obtained with the program STRUCTURE (Pritchard et al., 2000 [**[54]**]) for 20 populations of *F. Suspensa *based on nrDNA variation.** Each vertical bar represents an individual and its assignment proportion (not shown) into one of three (colored) population clusters (*K*).

Bayesian analysis based on nrDNA revealed a progressive increase in *L* (*K*) until *K* = 3 (data not shown). However, the resulting groupings did not correspond to separate geographical regions supported by cpDNA data (Figure 4). Non-hierarchical AMOVA and Nei’s estimator of population substructure (*G*_ST_) indicated high levels of population differentiation for nrDNA in *F. suspensa* at the species-range scale (*Φ*_ST_ = 0.207; Table 2). Despite taking into account the species’ weak hierarchical (regional) substructure (*Φ*_CT_ = −0.049), overall levels of population divergence still remained high (*Φ*_SC_ = 0.236; *P* < 0.001; Table 2). However, no significant isolation by geographical distance (*r* = −0.019, *P* > 0.05) and isolation by ecological distance (*r* = −0.046, *P* > 0.05) were found for nrDNA. Using an nrDNA-derived *Φ*_ST (*n*)_ value of 0.207 across all surveyed populations (see above) and the corresponding value of cpDNA (*Φ*_ST (*c*)_ = 0.929), the pollen/seed migration ratio (*r*) was calculated as 48.4, indicating that pollen flow was significantly higher than seed flow.

Tajima’s *D* and Fu’s *F*s statistics based on nrDNA data were examined for each of the seven groups. Shandong and HHS groups significantly deviated from neutrality. However, mismatch analysis did not support the demographic expansion of HHS group (Table 3).

### Climatic suitability inference by ecological niche modeling

**Figure 5:**
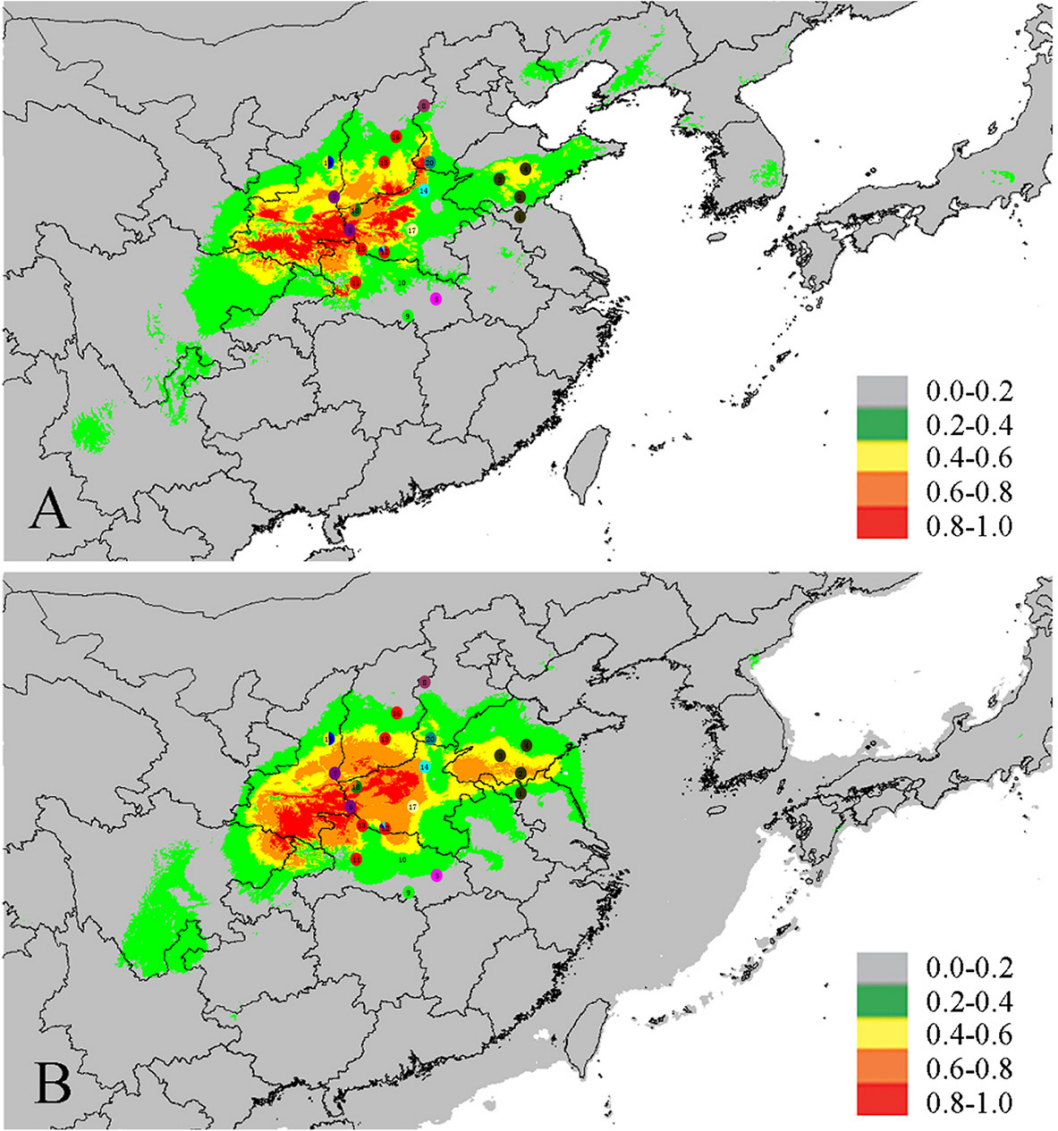
**Maps showing the bioclimatic suitability for *F. suspensa *through ecological niche modelling with Maxent using bioclimatic variables (grey, 0–0.2; green**, **0.2–0.4; yellow, 0.4–0.6; orange**, **0.6–0.8; red, 0.8–1.0).** Map obtained from software DIVA-GIS. **A**, the present, **B**, at the Last Glacial Maximum (LGM; 21 thousand years before present (ka)) based on the CCSM model.

The predicted areas climatically suitable for *F. suspensa* for the current and past (LGM) are illustrated in Figure 5. The values of AUC based on both training and test presence data for the present were all higher than expected by chance (0.995 and 0.998), demonstrating good model performance. Notably, the model indicated that *F. suspensa* experienced habitat fragmentation isolated by intervening unsuitable habitat, and significant isolation was found between the Shandong group and other groups (Figure 5A). The area of climatically suitability value above 0.4 from the LGM prediction (430,602 km^2^) was larger than that from the current prediction (311,860 km^2^). The area of climatically suitability value above 0.8 at LGM (70,032 km^2^) was also slightly larger than the current most suitable habitat (67,329 km^2^). Obviously climatically suitable areas expanded in the Shandong group, retreated in the northern edge (Hebei and Shandong provinces) and extended southward to the edge of subtropical Chongqing and Hubei provinces during the LGM (see Figure 5B).

## Discussion

### Genetic diversity and spatial population genetic structure

Generally, geographical distribution, breeding system, and population size all affect genetic diversity in plant species [27-29]. *F. suspensa* is an out-crossing species that has two mechanisms for avoiding autogamy, herkogamy and dichogamy [30]. *F. suspensa* is also a widespread species with a large population size. All these features should result in a relatively high genetic diversity of *F. suspensa*. However, the species-wide level of genetic diversity (*h*_T_ = 0.625) was unexpectedly lower than that of other seed plants in China based on the ITS, such as *Eriophyton wallichii* (*h*_T_ = 0.908) [31], *Achyranthes bidentata* (*h*_T_ = 0.703) [32], and *Primula obconica* (*h*_T_ = 0.994) [20]. The species-wide level of cpDNA-derived genetic diversity (*h*_T_ = 0.867) was moderate compared with 13 other seed plants used as maternally inherited markers in China [13]. Three possible factors contributed to the observed relatively low level of genetic diversity. First, the current climatically suitable areas predicted by the ENM suggested that *F. suspensa* may have experienced habitat fragmentation, which was probably the key contributory factor resulted in the loss of genetic diversity. Second, the low genetic diversity may be caused by increased human activity because this region had become a developed agricultural area since the Neolithic Period. Third, as a main Chinese traditional drug, over-harvesting of its fruit may have caused a sharp decline in the quantity of plants and may have also accelerated the loss of genetic diversity.

In addition, cpDNA data demonstrated significant population differentiation within *F. suspensa*. Population subdivision based on cpDNA data (*Φ*_ST_ = 0.929) was much greater than the average level of other seed plants for maternally inherited markers (mean *Φ*_ST_ = 0.670) [33]. Another striking feature of *F. suspensa* was the marked group differentiation based on cpDNA (*Φ*_CT_ = 0.793). Significant IBD pattern was indicated by cpDNA (*r* = 0.343; *P* < 0.001) analyses, suggesting that gene flow declined with increased geographical distance. Generally, gene flow estimations using plastid DNA markers are based on DNA transmission through seeds in most angiosperm species [34]. However, *F. suspensa* lacks efficient seed dispersal mechanisms, and seeds of *F. suspensa* are small with wing-like structures. The seeds are dispersed by wind, the dispersal distance is short, and most seeds are spread in the same population, which may be one of the main reasons for the high levels of genetic differentiation. In addition, environmental conditions in mountains at higher elevations markedly differed from those in the intervening plains, which probably acted as barriers to gene flow through seeds and further resulted in high levels of interpopulation genetic divergence. However, due to lack of long distance dispersal mechanism for *F. suspensa*, the significant genetic differentiation among populations didn’t be caused by the ecological distance. Meanwhile, considering the cultivated land area in low-elevation plains, the DBLFs in the warm-temperate zone of China were fragmented, which led to patch-like habitats of *F. suspensa*.

In contrast to the significant phylogeographical structure obtained with cpDNA, only a moderate level of genetic differentiation and phylogeographical structure was suggested by nrDNA when examined over all populations (*Φ*_ST_ = 0.207). Limited seed flow and high pollen flow among populations was considered to be the explanation for the nrDNA-derived population differentiation in *F. suspensa*. In fact, the pollen-to-seed migration ratio (*r*) obtained for *F. suspensa* (*r* = 48.4) was obviously higher than the corresponding average value reported for seed plant species (median *r* ≈ 17 estimated over 93 species) [33,35]. The spatial pattern of maternally inherited cpDNA differentiation has indicated the presence of obvious genetic structure; gene flow through long-distance dispersed pollen usually erodes the genetic signature of isolation by distance. Thus, our results represented another example of this situation and provided evidence of efficient pollen-mediated gene flow among the isolated populations. Considering the effects of such long-distance, pollen-mediated gene flow, forest fragmentation and habitat isolation among populations may not have played an important role in nuclear genomic diversification and speciation. However, analysis of isolation by geographical and ecological distance based on nrDNA data indicated geographical or ecological factor was not related to pollen-mediated gene flow, which may be due to the mutual interference among different gene pools (i.e., the seven groups identified by cpDNA).

### Inference of phylogeographical history in *F. suspensa*

Climatic changes during Pleistocene glacial cycles are believed to have affected the present distribution pattern and phylogeographical structure of plant species [4,36,37]. However, the role of these climatic fluctuations in China’s warm-temperate zone and their potential importance in the current population genetic structuring of regional vegetation is not well understood. In order to investigate the effection by climatic changes during Pleistocene glacial cycles, we selected *F. suspensa* as a model to test the three hypotheses which described in the introduction. In this work, the partitioning of genetic variability-based cpDNA had a significant geographical component, each group had its own unique chlorotype, and no chlorotype was shared among groups. Printzen et al., reported large-scale intraspecific disjunctions in many species that can alternatively be explained by range fragmentation and widespread long-distance dispersal [38]. Given the lack of effective seed dispersal mechanism, widespread long-distance dispersal may not be the main reason for intraspecific disjunctions [39]. Thus, the heterogeneous chlorotype composition and genetic structure may be ascribed to range fragmentation. Fragmentation may be a consequence of isolation that can either be geographical or environmental. However, no obvious geographical barrier was found among the seven groups. Therefore, *F. suspensa* did not appear to be geographically isolated, thereby allowing ecological niche modeling to be used in assessing species status. The model prediction for the current time indicated that *F. suspensa* may have experienced habitat fragmentation isolated by intervening unsuitable habitat. This phenomenon was particularly prominent between the Shandong group and other groups (Figure 5A). Our divergence time analysis revealed that the divergence of the species most likely fell into the Early-to-Late Pleistocene, about 2.70 Ma to 0.25 Ma, coinciding with frequent climatic oscillations during the Pleistocene, which is considered as one of the most important periods for genetic diversification and speciation [3,40]. Molecular clock analysis of chlorotype variation agreed with phylogenetic analyses in indicating the divergence of species long predating the LGM. Thus, we presumed that the subdivision of the seven groups resulted from allopatric fragmentation in the past.

To further infer demographic processes, two markers were used to detect each group. The apparent population expansion (inferred from the climatically suitable area expansion) was found only in the Shandong group, which was supported by the mismatch distribution, as well as Tajima’s *D* and Fu’s *Fs*, values based on nrDNA data (Table 3), was also supported by ecological niche modeling (Figure 5). However, population expansion in the Shandong group interestingly occurred not during warming stages but during glacial stages (Figure 5). Although apparent population expansion was not detected in most groups by demographic analyses, we still presumed that the other six groups (except for the Shandong group) experienced limited population expansion for high number of low-frequency private ribotype in most populations (Table 4). Similarly, these limited population expansion were also occurred during glacial stages, this opinion was also supported by ENM (Figure 5). Taken together, a scenario of repeated expanding to nearby low altitude plains in the glacial periods (similar to LGM, Figure 5B), and by retreating to mountaintops during interglacial warmer stages (similar to current days, Figure 5A) is the most likely for *F. suspensa* during the Quaternary climatic changes. However, population expansion did not result in continuous glacial distribution, and no geographical contact existed between groups. This suggestion was supported by the heterogeneous chlorotype composition of groups. Unlike cpDNA data, nrDNA data supported populations were clustered into three groups, with each group including samples from two or more separate cpDNA geographical groups (Figure 4). The discordance between the patterns revealed by cpDNA and nrDNA data indicated more extensive pollen-mediated gene flow than seed-mediated gene flow among the separate geographical groups.

By assuming *F. suspensa* agreed with the first hypothesis, we would expect to see a high diversity in southern populations and impoverished intrapopulation genetic diversity in the direction of recolonization [3,4,41]. However, our genetic analysis based on molecular dada did not support this view, i.e., the two southernmost populations P5 and P9 did not have a higher genetic diversity (Tables 1 and 4). Ecological niche modeling also suggested *F. suspensa* was not compressed and underwent long southward migration during the LGM. Under the scenario that the second hypothesis was appropriate for *F. suspensa*, present-day populations in the re-colonized area would probably show near genetic uniformity for derived chlorotypes and significant genetic diversity decline from refugia to the recolonized area. However, our data suggested that only the Shandong group had uniform chlorotype, and the other groups (i.e., HHS and HHS) had heterogeneous chlorotype. In addition, no significant longitudinal or latitudinal gradient in nrDNA diversity was observed in the three groups (i.e., Shandong, HHS, and HHS); the other four groups were not calculated because only one population existed. Based on analysis of isolation by ecological distance for cpDNA (*r* = −0.113; *P* > 0.05), which further indicated no long distance dispersal occurred for this species. Thus, most aspects of the herein characterized phylogeographical structure of *F. suspensa* agreed with the evolutionary model of interglacial compression and glacial-limited expansion. Although *F. suspensa* obviously retreated at the northern edge, no evidence was found for the long-distance southward migration or large-scale range contraction during glaciation.

## Conclusions

Analysis of molecular data and ecological niche modeling suggested that *F. suspensa* tracked Quaternary climatic changes by expanding to nearby low-elevation plains in the glacial periods and by retreating to mountaintops during interglacial warmer stages, thereby experiencing fragmentation and isolation. No geographical contact zone existed among groups during the Pleistocene glacial cycles, and extensive pollen-mediated gene flow weakened the genetic divergence among separate geographical groups.

## Methods

### Population sampling

Silica-dried samples of leaf material were obtained from 20 populations, representing almost the entire natural distribution area of *F. suspensa*. The distance between samples within populations was at least 10 m to increase the likelihood of sampling inter-individual variation within each population. Geographical information regarding these populations and numbers of individuals used in the cpDNA and nrDNA analyses are presented in Table 1.

### DNA isolation, polymerase chain reaction (PCR) amplification, and DNA sequencing

Total DNA was extracted from approximately 30 mg of dried leaf tissue using a Plant Genomic DNA Kit (TIANGEN, Beijing, China) according to the manufacturer’s protocol. For phylogeographical DNA surveys, we sequenced two regions of cpDNA (*trn*L-F [42], *psb*A-*trn*H [43]), including the internal transcribed spacer region (ITS) of nrDNA (*ITS*4-*ITS*5 [44]). PCR was performed in a reaction volume of 30 μL containing 30 ng of genomic DNA, 0.2 mmol/L of each dNTP, 0.3 μmol/L of each primer, 3 μL of Taq buffer, and 1 unit of Taq polymerase (Takara, Dalian, Liaoning, China). The PCR protocols involved initial denaturation for 4 min at 94°C followed by 35 cycles of 40 s at 94°C, 45 s at 50°C, 90 s at 72°C, and a final extension step of 8 min at 72°C. The PCR products were purified with an E.Z.N.A. Gel Extraction Kit (Omega Bio-Tek, Winooski, VT, USA) and then sequenced on an ABI 3730 DNA Sequence Analyzer at BGI (Beijing, China). Sequence quality was checked against the original chromatogram and aligned using CLUSTAL_X version 1.81 [45]. For ITS sequencing, the presence of “double peaks” at polymorphic sites in the chromatogram was manually checked. Haplotypes were first determined by “haplotype subtraction” [46,47].

### Molecular data analysis

Sequences for each fragment were aligned by CLUSTAL_X version 1.81 [45], and indels were coded as substitutions following the method of Caicedo & Schaal [48]. Haplotype diversity (*h*) and nucleotide diversity (*π*) were calculated for each population (*h*_S_ and *π*_S_) and at the species level (*h*_T_ and *π*_T_) using DNASP version 5.0 [49]. cpDNA haplotype networks were constructed in TCS version 1.21 [50], which showed all linkages between haplotypes with >95% probability of being most parsimonious. Phylogenetic analyses of cpDNA haplotype sequences were performed with MEGA5.2 [51] using the neighbor-joining method on Kimura 2-parameter distances [52]. Bootstrap values were estimated to assess the relative support for relationships between haplotypes (1000 replicates) [53]. These analyses included *Forsythia viridissima* as an outgroup. Determination of the divergence times of lineages within species can help elucidate the historic events involving the species. Considering the lack of reliable fossil record in genus *Forsythia*, the average divergence time was estimated via calibrating molecular clock implemented in MEGA 5.2 [51]. Therefore, the minimum and maximum values of a range of average mutation rates reported for synonymous sites of plant chloroplast genes [i.e. 1.2 and 1.7 × 10^−9^ substitutions per site per year (s/s/y)] were taken [26].

To infer the most likely number of population genetic clusters (*K*) in the cpDNA and nrDNA dataset, we performed Bayesian analysis of population structure as implemented in STRUCTURE version 2.2 [54]. *K* ranged from 1 to 10, with 10 replicates performed for each *K* and using a burn-in period of 2 × 10^5^ and 5 × 10^4^ Monte Carlo and Markov chains. The “no-admixture model” and independent allele frequencies were chosen for this analysis. The most likely number of clusters was identified using the maximal value of *L* (*K*) returned by STRUCTURE [54,55]. Genetic divergence among populations was inferred from Nei’s estimator [56] of population substructure (*G*_ST_) as well as from *Φ*_ST_ obtained from non-hierarchical analyses of molecular variance (AMOVAs) in ARLEQUIN version 3.5 [57]. Hierarchical AMOVA was also used to quantify the partitioning of cpDNA and nrDNA variance between regional groups of populations (*Φ*_CT_) and between populations within such groups (*Φ*_SC_). Significance levels of *Φ* statistics were based on 10 000 permutations [58].

For both nrDNA and cpDNA data, tests of isolation-by-distance (IBD) were performed by regressing values of *Φ*_ST_ against the geographical distance (*K*_m_) and the ecological distance (least cost distance) with the Mantel permutation procedure as implemented in IBD (Isolation by Distance Web Service: BMC Genetics 6: 13. v.3.16 http://ibdws.sdsu.edu/) [59]. According the method of Acevedo et al. [60], we computed the ecological distance between populations using the least-cost distance algorithm implemented in ArcGIS 10 [61]. This algorithm calculates a deterministic least-cost distance between a source population and a target population using a friction layer. Here, the friction map was obtained as one minus the predictions of the past model for the LGM [60]. A pollen/seed migration ratio (*r*) was calculated using a modified equation of Ennos [62], according to Petit et al. [33], with AMOVA-derived *Φ*_ST_ values (instead of *G*_ST_) taken as estimators of population differentiation: *r* = *m*_p_/*m*_s_ = [(1/*Φ*_ST (n)_ − 1) − 2(1/*Φ*_ST (c)_ − 1)]/(1/*Φ*_ST (c)_ − 1), where *m*_p_ is the pollen migration rate, *m*_s_ is the seed migration rate, *Φ*_ST (n)_ is the nuclear (nrDNA) *Φ*_ST_, and *Φ*_ST (c)_ is the cytoplasmic (cpDNA) *Φ*_ST_.

To determine whether the cpDNA and nrDNA sequences satisfied the assumption of neutrality, we calculated Tajima’s *D*[63] and Fu’s *F*s [64] values for the entire species and groups of populations using ARLEQUIN. Statistical significance of *D* and *Fs* was estimated with coalescent simulations as implemented in this program. Generally, a significantly negative Tajima’s *D* indicates an excess of low-frequency alleles that can arise from purifying selection, rapid population expansion, and selective sweeps [65]. Fu’s *Fs*[64] is expected to have significantly large and negative values under conditions of demographic expansion. To further infer demographic processes, we explicitly tested the null hypotheses of the sudden expansion model in ARLEQUIN by comparing observed and expected distributions of pairwise sequence differences (mismatch distributions). The sum of squared deviations (SSD) and raggedness index (RAG) were used to test the goodness-of-fit of the observation mismatch distribution to the expectation model.

### Past and current climatic suitability inferences

To investigate the effect of cold periods (such as during the LGM) on climatic suitability of *F. suspensa*, we inferred the climatically suitable areas using ecological niche models. Assuming that the species did not change climatic preference (viz, niche conservatism [66]), the climatically suitable areas of *F. suspensa* at the LGM were reconstructed according to the current distribution by implementing a maximum entropy model Maxent 3.1.0 [67]. Maxent software is considered to be more robust than other methods for predicting species distributions [68,69]. Information on the geographic distribution of *F. suspensa* was based on a set of 50 presence points covering the entire distribution range of *F. suspensa*: 30 points were obtained from the Chinese Virtual Herbarium (http://www.cvh.org.cn/cms/) and 20 points were from sampling sites (Additional file 3). Current bioclimatic variables and LGM data were downloaded from the WorldClim database (http://www.worldclim.org/). For climate layers we used bioclimatic variables at 2.5 arc-minute resolution [70]. Current conditions were based on the observed data, representative of 1950–2000. LGM data were simulated using the Community Climate System Model [71]. All of 19 bioclimatic variables in the WorldClim database were used to model climatic suitability for the species. The area selected to calibrate ENMs has effects on the predictive performance of the models [72]. It should be large enough to account for the full response of the species to the climatic gradients [72,73]. Since the focal species *F. suspensa* is a species endemic to China’s Warm Temperate Zone, a set of 50 presence points used in this study covers most distribution areas of this species. We selected an area that covers this zone to calibrate the model, and the predictions for both the current and LGM were also made for this area.

To construct ENMs, we used the default parameters of MAXENT and the following user-selected features: regularization multiplier of 1.0, application of a random seed, duplicate presence records removal and cumulative probabilities used for the output. To test the performance of each model, 20% of the data in each run was randomly selected by MAXENT and compared with the model output generated with the remaining data. The area under the receiver operating characteristic curve (AUC) was used to assess model performance [67].

### Availability of supporting data

The data sets supporting the results of this article are available in the Dryad Digital Repository: http://doi.org/10.5061/dryad.f5c60.

## Competing interests

The authors declare that they have no competing interests.

## Authors’ contributions

YL conceived the research project; KMZ, YL, YHL and ZZF collected the data, YHL, Y L and ZZF analysed the data; YL and ZZF wrote the manuscript. All authors read and approved the final manuscript.

## Supplementary Material

Additional file 1 **Chloroplast DNA sequence polymorphisms detected in two intergenic spacer (IGS) regions of *F. suspensa *** **identifying thirteen chlorotypes (C1–C13).** All sequences are relative to the reference haplotype C1. Numbers 1/0 in sequences denote presence/absence of length polymorphism, identified by superscript letter (a, b, c). Click here for file

Additional file 2 **Nuclear DNA sequence polymorphisms detected in internal transcribed spacer (ITS) regions of *F. suspensa *** **identifying seventy-four ribotypes (R1–R74).** All sequences are relative to the reference haplotype R1. Click here for file

Additional file 3 **Geographic characteristics of 50** ***F. suspensa *** **presence points used in this study.** Click here for file

## References

[B1] Hewitt GM Some genetic consequences of ice ages, and their role in divergence and speciation Biol J Linn Soc 1996 58 3 247 276 10.1111/j.1095-8312.1996.tb01434.x

[B2] Comes HP Kadereit JW The effect of Quaternary climatic changes on plant distribution and evolution Trends Plant Sci 1998 3 11 432 438 10.1016/S1360-1385(98)01327-2

[B3] Hewitt GM The genetic legacy of the Quaternary ice ages Nature 2000 405 6789 907 913 10.1038/35016000 10879524

[B4] Hewitt GM Genetic consequences of climatic oscillations in the Quaternary Philos Trans Roy Soc Lond B Biol Sci 2004 359 1442 183 195 10.1098/rstb.2003.1388 15101575 PMC1693318

[B5] Hewitt GM Rothschild LJ, Lister AM Ice ages: their impact on species distributions and evolution Evolution on Planet Earth 2003 Oxford: Academic Press 339 361

[B6] Petit RJ Aguinagalde I de Beaulieu JL Bittkau C Brewer S Cheddadi R Ennos R Fineschi S Grivet D Lascoux M Glacial refugia: hotspots but not melting pots of genetic diversity Science 2003 300 5625 1563 1565 10.1126/science.1083264 12791991

[B7] Avise JC Phylogeography: retrospect and prospect J Biogeogr 2009 36 1 3 15 10.1111/j.1365-2699.2008.02032.x

[B8] Hickerson MJ Carstens BC Cavendar-Bares J Crandall KA Graham CH Johnson JB Rissler L Victoriano PF Yoder AD Phylogeography’s past, present and future: 10 years after Avise, 2000 Mol Phylogenet Evol 2010 54 1 291 301 10.1016/j.ympev.2009.09.016 19755165

[B9] Shi YF Cui ZJ Su Z The Quaternary glaciations and enviromental changes in China 2006 Shijiazhuang: Hebei Science and Technology Publishing Press

[B10] Li XH Shao JW Lu C Zhang XP Qiu YX Chloroplast phylogeography of a temperate tree *Pteroceltis tatarinowii* (Ulmaceae) in China J Syst Evol 2012 50 4 325 333 10.1111/j.1759-6831.2012.00203.x

[B11] Yu G Chen X Ni J Cheddadi R Guiot J Han H Harrison SP Huang C Ke M Kong Z Li S Li W Liew P Liu G Liu J Liu Q Liu KB Prentice IC Qui W Ren G Song C Sugita S Sun X Tang L van Campo E Xia Y Xu Q Yan S Yang X Zhao J Palaeovegetation of China: a pollen data based synthesis for the mid-Holocene and last glacial maximum J Biogeogr 2000 27 3 635 664 10.1046/j.1365-2699.2000.00431.x

[B12] Harrison SP Yu G Takahara H Prentice IC Palaeovegetation: Diversity of temperate plants in East Asia Nature 2001 413 6852 129 130 10.1038/35093166 11557970

[B13] Qiu YX Fu CX Comes HP Plant molecular phylogeography in China and adjacent regions: Tracing the genetic imprints of Quaternary climate and environmental change in the world’s most diverse temperate flora Mol Phylogenet Evol 2011 59 1 225 244 10.1016/j.ympev.2011.01.012 21292014

[B14] Liu JQ Sun YS Ge XJ Gao LM Qiu YX Phylogeographic studies of plants in China: Advances in the past and directions in the future J Syst Evol 2012 50 4 267 275 10.1111/j.1759-6831.2012.00214.x

[B15] Gao LM Moller M Zhang XM Hollingsworth ML Liu J Mill RR Gibby M Li DZ High variation and strong phylogeographic pattern among cpDNA haplotypes in *Taxus wallichiana* (Taxaceae) in China and North Vietnam Mol Ecol 2007 16 22 4684 4698 10.1111/j.1365-294X.2007.03537.x 17908214

[B16] Wang HW Ge S Phylogeography of the endangered *Cathaya argyrophylla* (Pinaceae) inferred from sequence variation of mitochondrial and nuclear DNA Mol Ecol 2006 15 13 4109 4122 10.1111/j.1365-294X.2006.03086.x 17054506

[B17] Wang J Gao P Kang M Lowe AJ Huang H Refugia within refugia: The case study of a canopy tree *Eurycorymbus cavalerieiin* subtropical China J Biogeogr 2009 36 11 2156 2164 10.1111/j.1365-2699.2009.02165.x

[B18] Su YJ Wang T Zheng B Jiang Y Chen GP Ouyang PY Sun YF Genetic differentiation of relictual populations of *Alsophila spinulosa* in southern China inferred from cpDNA *trn*L-F noncoding sequences Mol Phylogenet Evol 2005 34 2 323 333 10.1016/j.ympev.2004.10.016 15619445

[B19] Tian S López-Pujol J Wang H Ge S Zhang ZY Molecular evidence for glacial expansion and interglacial retreat during Quaternary climatic changes in a montane temperate pine (*Pinus kwangtungensis* Chun ex Tsiang) in southern China Plant Syst Evol 2010 284 3–4 219 229

[B20] Yan HF Zhang CY Wang FY Hu CM Ge XJ Hao G Population expanding with the phalanx model and lineages split by environmental heterogeneity: a case study of *Primula obconicain* Subtropical China Plos one 2012 7 9 e41315 10.1371/journal.pone.0041315 23028425 PMC3446961

[B21] Gao XM Ma KP Chen LZ Species diversity of some deciduous broad-leaved forests in the warm-temperate zone and its relations to community stability Acta Phytoecol Sin 2001 25 3 283 290

[B22] Zhu H Notes on the origin of temperate deciduous broad-leaved forests of East Asia Bull Bot Res 1997 17 4 388 396

[B23] Chen SC Zhang L Zeng J Shi F Yang H Mao YR Fu CX Geographic variation of chloroplast DNA in *Platycarya strobilacea* (Juglandaceae) J Syst Evol 2012 50 4 374 385 10.1111/j.1759-6831.2012.00210.x

[B24] Qi XS Chen C Comes HP Sakaguchi S Liu YH Tanaka N Sakiom H Qiu YX Molecular data and ecological niche modelling reveal a highly dynamic evolutionary history of the East Asian Tertiary relict *Cercidiphyllum* (Cercidiphyllaceae) New Phytol 2012 196 2 617 630 10.1111/j.1469-8137.2012.04242.x 22845876

[B25] Zhao C Wang CB Ma XG Liang QL He XJ Phylogeographic analysis of a temperate-deciduous forest restricted plant (*Bupleurum longiradiatum* Turcz.) reveals two refuge areas in China with subsequent refugial isolation promoting speciation Mol Phylogenet Evol 2013 68 3 628 643 10.1016/j.ympev.2013.04.007 23624194

[B26] Graur D Li WH Fundamentals of Molecular Evolution 2000 2 Sunderland Massachusetts: Sinauer Associates press

[B27] Hamrick JL Godt MJW Sherman-Broyles SL Factors influencing levels of genetic diversity in woody plant species New For 1992 6 1–4 95 124

[B28] Hamrick JL Godt MJW Avise JC, Hamrick JL Conservation genetics of endemic plant species Conservation Genetics 1996 New York: Chapman and Hall 281 304

[B29] Nybom H Comparison of different nuclear DNA markers for estimating intraspecific genetic diversity in plants Mol Ecol 2004 13 5 1143 1155 10.1111/j.1365-294X.2004.02141.x 15078452

[B30] Li JY Zhang ZX Yi WY Flower structure and reproduction system of *Forsythia suspensa* Vahl Acta Bot Boreal-Occident Sin 2006 26 8 1548 1553

[B31] Wang XX Yue JP Sun H Li ZM Phylogeographical study on *Eriophyton wallichii* (Labiatae) from alpine scree of Qinghai Tibetan Plateau Plant Divers Resour 2011 33 6 605 614

[B32] Li Y Liu P Li YH Intraspecific variation of *Achyranthes bidentata* (Amaranthaceae) in the geo-authentic product area based on internal transcribed spacer sequences of ribosomal DNA Aust J Crop Sci 2012 6 12 1655 1660

[B33] Petit RJ Duminil J Fineschi S Salvini D Vendramin GG Comparative organization of chloroplast, mitochondrial and nuclear diversity in plant populations Mol Ecol 2005 14 3 689 701 15723661 10.1111/j.1365-294X.2004.02410.x

[B34] Petit RJ Vendramin GG Weiss S, Ferrand N Phylogeography of organelle DNA in plants: an introduction Phylogeography of southern European Refugia 2007 New York: Springer 23 97

[B35] Hodgins KA Barrett SCH Population structure and genetic diversity in tristylous *Narcissus triandrus*: Insights from microsatellite and chloroplast DNA variation Mol Ecol 2007 16 11 2317 2332 10.1111/j.1365-294X.2007.03314.x 17561893

[B36] Petit RJ Grivet D Optimal randomization strategies when testing the existence of a phylogeographic structure Genetics 2002 161 1 469 471 12051195 10.1093/genetics/161.1.469 PMC1462084

[B37] Bartish IV Kadereit JW Comes HP Late Quaternary history of *Hippophae rhamnoides* L. (Elaeagnaceae) inferred from chalcone synthase intron (*Chsi*) sequences and chloroplast DNA variation Mol Ecol 2006 15 13 4065 4083 10.1111/j.1365-294X.2006.03079.x 17054503

[B38] Printzen C Ekman S Tonsberg T Phylogeography of *Cavernularia hultenii*: evidence of slow genetic drift in a widely disjunct lichen Mol Ecol 2003 12 6 1473 1486 10.1046/j.1365-294X.2003.01812.x 12755876

[B39] Ma SM Zhang ML Sanderson SC Phylogeography of the rare *Gymnocarpos przewalskii* (Caryophyllaceae): indications of multiple glacial refugia in north-western China Aust J Bot 2012 60 1 20 31 10.1071/BT11055

[B40] Willis KJ Van Andel TH Trees or no trees? The environments of central and eastern Europe during the Last Glaciation Quaternary Sci Rev 2004 23 23–24 2369 2387

[B41] Petit RJ Hu FS Dick CW Forests of the past: a window to future changes Science 2008 320 5882 1450 1452 10.1126/science.1155457 18556547

[B42] Taberlet P Gielly L Pautou G Bouvet J Universal primers for amplification of three noncoding regions of chloroplast DNA Plant Mol Biol 1991 17 5 1105 1109 10.1007/BF00037152 1932684

[B43] Kress WJ Wurdack KJ Zimmer EA Weigt LA Janzen DH Use of DNA barcodes to identify flowering plants Proc Natl Acad Sci U S A 2005 102 23 8369 8374 10.1073/pnas.0503123102 15928076 PMC1142120

[B44] White TJ Bruns T Lee S Taylor J Innis MS, Gelfand DH, Sninsky JJ, White TJ Amplification and direct sequencing of fungal ribosomal RNA genes for phylogenetics PCR protocols: a guide to methods and applications 1990 San Diego: Academic Press 315 322

[B45] Thompson JD Gibson TJ Plewniak F Jeanmougin F Higgins DG The ClustalX windows interface: Flexible strategies for multiple sequence alignment aided by quality analysis tools Nucleic Acids Res 1997 25 24 4876 4882 10.1093/nar/25.24.4876 9396791 PMC147148

[B46] Clark AG Inference of haplotypes from PCR-amplified samples of diploid populations Mol Biol Evol 1990 7 2 111 122 2108305 10.1093/oxfordjournals.molbev.a040591

[B47] Zhou RC Zeng K Wu W Chen XS Yang ZH Shi SH Wu CI Population genetics of speciation in non-model organisms: I. Ancestral polymorphism in mangroves Mol Biol Evol 2007 24 12 2746 2754 10.1093/molbev/msm209 17906000

[B48] Caicedo AL Schaal BA Population structure and phylogeography of *Solanum pimpinellifolium* inferred from a nuclear gene Mol Ecol 2004 13 7 1871 1882 10.1111/j.1365-294X.2004.02191.x 15189210

[B49] Librado P Rozas J DnaSP v5: A software for comprehensive analysis of DNA polymorphism data Bioinformatics 2009 25 11 1451 1452 10.1093/bioinformatics/btp187 19346325

[B50] Clement M Posada D Crandall KA TCS: A computer program to estimate gene genealogies Mol Ecol 2000 9 10 1657 1660 10.1046/j.1365-294x.2000.01020.x 11050560

[B51] Tamura K Peterson D Peterson N Stecher G Nei M Kumar S MEGA5: Molecular evolutionary genetics analysis using maximum likelihood, evolutionary distance, and maximum parsimony methods Mol Biol Evol 2011 28 10 2731 2739 10.1093/molbev/msr121 21546353 PMC3203626

[B52] Kimura M A simple method for estimating evolutionary rates of base substitutions through comparative studies of nucleotide sequences J Mol Evol 1980 16 2 111 134 10.1007/BF01731581 7463489

[B53] Felsenstein J Confidence limits on phylogenies: an approach using the bootstrap Evolution 1985 39 4 783 791 10.2307/2408678 28561359

[B54] Pritchard JK Stephens M Donnelly P Inference of population structure using multilocus genotype data Genetics 2000 155 2 945 959 10835412 10.1093/genetics/155.2.945 PMC1461096

[B55] Evanno G Regnaut S Goudet J Detecting the number of clusters of individuals using the software structure: A simulation study Mol Ecol 2005 14 8 2611 2620 10.1111/j.1365-294X.2005.02553.x 15969739

[B56] Nei M Analysis of gene diversity in subdivided populations Proc Natl Acad Sci U S A 1973 70 12 3321 3323 10.1073/pnas.70.12.3321 4519626 PMC427228

[B57] Excoffier L Lischer HEL Arlequin suite ver 3.5: A new series of programs to perform population genetics analyses under Linux and Windows Mol Ecol Resour 2010 10 3 564 567 10.1111/j.1755-0998.2010.02847.x 21565059

[B58] Excoffier L Smouse PE Quattro JM Analysis of molecular variance inferred from metric distances among DNA haplotypes: Application to human mitochondrial DNA restriction data Genetics 1992 131 2 479 491 1644282 10.1093/genetics/131.2.479 PMC1205020

[B59] Jensen JL Bohonak AJ Kelley ST Isolation by distance, web service BMC Genet 2005 6 13 15760479 10.1186/1471-2156-6-13 PMC1079815

[B60] Acevedo P Melo-Ferreira J Real R Alves PC Past, present and future distributions of an Iberian Endemic, *Lepus granatensis*: ecological and evolutionary clues from species distribution models PLoS One 2012 7 12 e51529 10.1371/journal.pone.0051529 23272115 PMC3521729

[B61] ESRI ArcGIS Desktop: Release 10 2011 Redlands, CA: Environmental Systems Research Institute

[B62] Ennos RA Estimating the relative rates of pollen and seed migration among plant populations Heredity 1994 72 3 250 259 10.1038/hdy.1994.35

[B63] Tajima F Statistical method for testing the neutral mutation hypothesis by DNA polymorphism Genetics 1989 123 3 585 595 2513255 10.1093/genetics/123.3.585 PMC1203831

[B64] Fu YX Statistical tests of neutrality of mutations against population growth, hitchhiking and background selection Genetics 1997 47 2 915 925 9335623 10.1093/genetics/147.2.915 PMC1208208

[B65] Chiang YC Schaal BA Ge XJ Chiang TY Range expansion leading to departures from neutrality in the nonsymbiotic hemoglobin gene and the cpDNA *trn*L-*trn*F intergenic spacer in *Trema dielsiana* (Ulmaceae) Mol Phylogenet Evol 2004 31 3 929 942 10.1016/j.ympev.2003.09.017 15120391

[B66] Peterson AT Soberón J Sánchez-Cordero V Conservatism of ecological niches in evolutionary time Science 1999 285 5431 1265 1267 10.1126/science.285.5431.1265 10455053

[B67] Phillips SJ Anderson RP Schapire RE Maximum entropy modeling of species geographic distributions Ecol Model 2006 190 3–4 231 259

[B68] Flanders J Li W Stephen JR Zhang SY Identifying the effects of the Pleistocene on the greater horseshoe bat, *Rhinolophus ferrumequinum*, in East Asia using ecological niche modelling and phylogenetic analyses J Biogeogr 2011 38 3 439 452 10.1111/j.1365-2699.2010.02411.x

[B69] Elith J Graham CH Anderson RP Dudík M Ferrier S Guisan A Hijmans RJ Huettmann F Leathwick JR Lehmann A Li J Lohmann LG Loiselle BA Manion G Moritz C Nakamura M Nakazawa Y McC J Overton M Peterson AT Phillips SJ Richardson K Scachetti-Pereira R Schapire RE Soberón J Williams S Wisz MS Zimmermann NE Novel methods improve prediction of species’ distributions from occurrence data Ecography 2006 29 2 129 151 10.1111/j.2006.0906-7590.04596.x

[B70] Hijmans RJ Cameron SE Parra JL Jones PG Jarvis A Very high resolution interpolated climate surfaces for global land areas Int J Climatol 2005 25 15 1965 1978 10.1002/joc.1276

[B71] Collins WD Bitz CM Blackmon ML Bonan GB Bretherton CS Carton JA Chang P Doney SC Hack JJ Henderson TB Kiehl JT Large WG McKenna DS Santer BD Smith RD The community climate system model version 3 (CCSM3) J Clim 2006 19 11 2122 2143 10.1175/JCLI3761.1

[B72] Anderson RP Raza A The effect of the extent of the study region on GIS models of species geographic distributions and estimates of niche evolution: preliminary tests with montane rodents (genus *Nephelomys*) in Venezuela: Effect of study region on models of distributions J Biogeogr 2010 37 7 1378 1393 10.1111/j.1365-2699.2010.02290.x

[B73] VanDerWal J Shoo LP Graham G Williams SE Selecting pseudo-absence data for presence-only distribution modeling: How far should you stray from what you know? Ecol Model 2009 220 4 589 594 10.1016/j.ecolmodel.2008.11.010

