# Supplementary material for: Molecular data and ecological niche modeling reveal population dynamics of widespread shrub Forsythia suspensa (Oleaceae) in China’s warm-temperate zone in response to climate change during the Pleistocene
Source: BMC Evol Biol. 2014 May 30;14:114. doi: 10.1186/1471-2148-14-114 (PMC4052925; doi:10.1186/1471-2148-14-114)
Supplement: Additional file 1 — Chloroplast DNA sequence polymorphisms detected in two intergenic spacer (IGS) regions of F. suspensa identifying thirteen chlorotypes (C1–C13). All sequences are relative to the reference haplotype C1. Numbers 1/0 in sequences denote presence/absence of length polymorphism, identified by superscript letter (a, b, c). [file 1471-2148-14-114-S1.pdf]

Additional file 1 Chloroplast DNA sequence polymorphisms detected in two intergenic spacer (IGS) regions of *F. suspensa* identifying thirteen chlorotypes (C1–C13). All sequences are relative to the reference haplotype C1. Numbers 1/0 in sequences denote presence/absence of length polymorphism, identified by superscript letter (a, b, c).

	Nucleotide position																		
	trnL-F						*psbA-trnH*												
Chlorotype	80	177	477	520	575	721	787	801	803	805	846	868	926	951	979	1011	1087	1112	1152
C1	1[a]	G	T	G	G	A	T	C	C	C	1[b]	0	C	G	C	C	C	T	C
C2	1[a]	G	C	G	G	A	T	C	C	C	1[b]	0	C	G	C	C	C	T	C
C3	1[a]	G	C	G	G	A	T	C	C	C	1[b]	0	C	G	C	C	C	C	C
C4	1[a]	G	C	G	G	A	T	T	C	C	1[b]	1[c]	C	G	C	C	C	C	C
C5	1[a]	G	C	G	G	A	T	C	C	C	1[b]	0	A	G	C	C	C	C	C
C6	1[a]	A	C	G	G	A	T	C	T	C	1[b]	0	A	G	C	C	C	C	C
C7	0	G	C	G	G	A	G	C	C	C	1[b]	0	A	G	T	C	C	C	C
C8	1[a]	G	C	G	G	A	T	C	C	C	1[b]	0	A	A	C	T	C	C	C
C9	1[a]	G	C	G	G	A	T	C	C	T	0	0	A	G	C	T	C	C	C
C10	1[a]	G	C	G	C	A	T	C	C	C	1[b]	0	A	G	C	T	C	C	C
C11	1[a]	G	C	C	G	A	T	C	C	C	1[b]	0	A	G	C	C	T	C	A

C12	1[a]	G	C	G	G	A	T	C	C	C	1[b]	0	A	G	C	C	C	C	A
C13	1[a]	G	C	G	G	G	T	C	C	C	1[b]	0	A	G	C	C	C	C	A

a, c: A; b: CTTTTTAATTACTACTACTTTATTTT
