# Supplementary material for: Molecular data and ecological niche modeling reveal population dynamics of widespread shrub Forsythia suspensa (Oleaceae) in China’s warm-temperate zone in response to climate change during the Pleistocene
Source: BMC Evol Biol. 2014 May 30;14:114. doi: 10.1186/1471-2148-14-114 (PMC4052925; doi:10.1186/1471-2148-14-114)
Supplement: Additional file 2 — Nuclear DNA sequence polymorphisms detected in internal transcribed spacer (ITS) regions of F. suspensa identifying seventy-four ribotypes (R1–R74). All sequences are relative to the reference haplotype R1. [file 1471-2148-14-114-S2.pdf]

Additional file 2 Nuclear DNA sequence polymorphisms detected in internal transcribed spacer (ITS) regions of *F. suspensa* identifying seventy-four ribotypes (R1–R74). All sequences are relative to the reference haplotype R1.

	ITS																																																													
Ribotype	11	24	27	43	44	51	55	58	59	60	65	68	70	71	75	93	94	96	98	101	108	112	118	195	237	242	276	280	290	291	297	298	299	300	301	302	304	313	317	318	322	324	327	338	343	350	351	357	358	362	375	388	390	395	397	414	415	421	446	448	451	461	476
R1	G	G	T	C	T	T	C	C	C	T	C	C	C	C	C	G	T	A	G	G	G	T	C	G	G	C	G	C	T	C	C	C	C	C	C	C	A	C	A	G	C	G	C	C	C	C	A	T	G	T	G	C	C	C	C	T	G	A	G	G	G	G	T
R2	G	G	T	C	T	T	C	C	C	T	C	C	C	C	C	G	T	A	G	G	G	T	C	G	G	C	G	C	T	C	C	T	C	C	C	C	A	C	A	G	C	G	C	C	C	C	A	T	G	T	G	C	C	C	C	T	G	A	G	G	G	G	T
R3	G	G	T	C	T	T	C	C	C	T	C	C	C	C	C	G	T	A	G	G	C	T	C	G	G	C	G	C	T	C	C	C	C	C	C	C	A	C	A	G	C	G	C	C	C	C	A	T	G	T	G	C	C	C	C	T	G	A	G	A	G	G	T
R4	G	G	T	C	T	T	C	C	C	C	C	C	C	C	C	G	T	A	G	G	G	T	C	G	G	C	G	C	T	C	C	C	C	C	C	C	A	C	A	G	C	G	C	C	C	G	A	T	G	T	G	C	C	C	C	T	G	A	G	G	G	G	T
R5	G	G	T	C	T	T	C	C	C	C	C	C	C	C	C	G	T	A	G	G	G	T	C	G	G	C	G	C	T	C	C	C	C	C	C	C	A	C	A	G	C	G	C	C	C	C	A	T	G	T	G	C	C	C	C	T	G	A	G	G	G	G	T
R6	G	G	T	C	T	T	C	C	C	T	C	C	C	C	C	G	T	A	G	G	G	T	C	G	G	C	G	C	T	C	C	T	C	T	C	C	A	C	A	G	C	G	C	C	C	C	A	T	G	T	G	C	C	C	C	T	G	A	G	G	G	G	T
R7	G	G	T	T	T	T	C	C	C	T	C	C	C	C	C	G	T	A	G	G	G	T	C	G	G	C	G	C	T	C	C	T	C	T	C	C	A	C	A	G	C	G	C	C	C	C	A	T	G	T	G	C	C	C	C	T	G	A	G	G	G	G	T
R8	G	G	T	C	T	T	C	C	C	T	C	C	C	C	T	G	T	A	G	G	G	T	C	G	G	C	G	C	T	C	C	T	C	T	C	C	A	C	A	G	C	G	C	C	C	C	A	T	G	T	G	C	C	C	C	T	G	A	G	G	G	G	T
R9	G	G	T	C	T	T	C	C	C	T	T	C	C	C	C	G	T	A	G	G	G	T	C	G	G	C	G	C	T	C	C	T	C	T	C	C	A	C	A	G	C	G	C	C	C	C	A	T	G	T	G	C	C	C	C	T	G	A	G	G	G	G	T
R10	G	G	T	C	T	T	C	C	C	T	T	C	C	C	C	G	T	A	G	G	G	T	C	G	G	C	G	C	T	C	C	T	C	C	C	C	A	C	A	G	C	G	C	C	C	C	A	T	A	T	G	C	C	C	C	T	G	A	G	G	G	G	T
R11	G	G	T	C	T	C	C	C	C	C	T	C	C	C	C	G	T	A	G	G	G	T	C	G	G	C	G	C	T	C	C	C	C	C	C	C	A	C	A	G	C	G	C	C	C	G	A	T	G	T	G	C	C	C	C	T	G	A	G	G	G	G	T
R12	G	G	T	C	T	T	C	C	C	C	T	C	C	C	C	G	T	A	G	G	G	T	C	A	G	C	G	C	T	C	C	C	C	C	C	C	A	C	A	G	C	G	C	C	C	C	A	T	G	T	G	C	C	C	C	T	G	A	G	G	G	G	T
R13	G	G	T	C	T	C	C	C	C	C	C	C	C	C	C	G	T	A	G	G	G	T	C	G	G	C	G	C	T	C	C	C	C	C	C	C	A	C	A	G	C	G	C	C	C	C	A	T	G	T	G	C	C	C	C	T	G	A	G	G	G	G	T
R14	G	G	T	C	T	T	C	C	C	T	C	C	C	C	C	G	T	A	G	G	G	T	T	G	G	C	G	C	T	C	C	T	C	C	C	C	A	C	A	G	C	G	C	C	C	C	A	T	G	T	G	C	C	C	C	T	G	A	G	G	G	G	T
R15	G	G	T	C	T	C	C	C	C	T	C	C	C	C	C	G	T	A	G	G	G	T	C	G	G	C	G	C	T	C	C	T	C	C	C	C	A	C	A	G	C	G	C	C	T	C	A	T	G	T	G	C	C	C	C	T	G	A	G	G	G	G	T
R16	G	G	T	C	T	C	C	C	C	C	C	C	C	C	C	G	T	A	G	G	G	T	C	G	G	C	G	C	T	C	C	T	C	C	C	C	A	C	A	G	C	G	C	C	C	C	A	T	G	T	G	C	C	C	C	T	G	A	G	G	G	G	T
R17	G	G	T	C	T	T	C	C	C	T	C	C	C	C	C	G	T	A	G	G	G	T	C	G	G	C	G	C	T	C	C	T	C	C	C	C	A	C	A	G	C	G	C	C	T	C	A	T	G	T	G	C	C	C	C	T	G	A	G	G	G	G	T
R18	G	G	T	C	T	C	C	C	C	C	C	C	C	C	C	G	T	A	G	G	G	T	C	G	G	T	G	C	C	C	C	C	T	C	C	C	A	C	A	G	C	G	C	C	C	C	A	T	G	T	G	C	C	C	C	T	G	A	G	G	G	G	T
R19	G	G	T	C	T	C	C	T	C	C	C	C	C	C	C	G	T	A	G	G	G	T	C	G	G	C	G	C	T	C	C	C	C	C	C	C	A	C	A	G	C	G	C	C	C	G	A	T	G	T	G	T	C	C	C	T	G	A	G	G	G	G	T
R20	G	G	T	C	T	C	C	C	C	C	C	C	C	C	C	G	T	A	G	G	G	T	C	G	G	C	G	C	C	C	C	C	T	C	C	C	A	C	A	G	C	G	C	C	C	C	A	T	G	T	G	C	C	C	C	T	G	A	G	G	G	G	T
R21	G	G	T	C	T	C	C	C	C	C	C	C	C	C	C	G	T	A	G	G	G	T	C	G	G	C	G	C	T	C	C	C	C	C	C	C	A	C	T	G	C	G	C	C	C	C	A	T	G	T	G	C	C	C	C	T	G	A	G	G	G	G	T
R22	G	G	T	C	T	C	C	C	C	C	C	C	C	C	C	G	T	A	G	G	G	T	C	G	G	C	G	C	T	C	C	C	C	T	C	C	A	C	T	G	C	G	C	C	C	C	A	T	G	T	G	C	C	C	C	T	G	A	G	G	G	G	T
R23	G	G	T	C	T	C	C	C	T	C	C	C	C	C	C	G	T	A	G	G	G	T	C	G	G	C	A	C	T	C	C	C	C	C	C	C	A	C	A	G	C	G	T	C	C	C	A	T	G	T	G	C	C	C	C	T	G	A	G	G	G	G	T
R24	G	G	T	C	T	C	C	C	T	C	C	C	C	C	C	G	T	A	G	G	G	T	C	G	G	C	G	C	C	C	C	C	T	C	C	C	A	C	A	G	C	G	C	C	C	C	A	T	G	T	G	C	C	C	C	T	G	A	G	G	G	G	T
R25	G	G	T	T	T	T	C	C	C	T	C	C	C	C	C	G	T	A	G	G	G	T	C	G	G	C	G	C	T	C	C	T	C	C	C	C	A	C	A	G	C	G	C	C	C	C	A	T	G	T	G	C	C	C	C	T	G	A	G	G	G	G	T
R26	G	G	T	C	T	T	C	C	C	T	C	C	C	C	C	G	T	A	G	G	G	T	C	G	G	C	G	C	T	C	C	T	C	C	C	C	A	C	A	G	C	G	C	C	C	C	A	T	G	T	G	C	C	C	C	T	G	A	A	G	G	G	T
R27	G	G	T	C	T	T	C	C	C	T	C	C	C	C	C	G	T	A	G	G	G	T	C	G	G	C	G	C	T	C	C	T	C	C	T	C	A	C	A	G	C	G	C	C	C	C	A	T	G	T	G	C	C	C	C	T	G	A	G	G	G	G	T
R28	G	G	T	C	T	T	C	C	T	T	C	C	C	C	C	G	T	A	G	G	G	T	C	G	G	C	G	C	T	C	C	C	C	C	C	C	A	C	A	G	C	G	T	C	C	C	A	T	G	T	G	C	C	C	C	T	G	A	G	G	G	G	T
R29	G	G	T	C	T	T	C	C	C	T	C	T	C	C	C	G	T	A	G	G	G	T	C	G	G	C	G	C	T	C	C	T	C	T	C	C	A	C	A	G	C	G	C	C	C	C	A	T	G	T	G	C	C	T	C	T	G	A	G	G	G	G	T
R30	G	G	T	C	T	T	C	C	C	T	C	C	C	C	C	G	T	A	G	G	G	T	C	G	G	C	G	C	T	C	C	T	C	T	C	C	A	C	A	G	C	G	C	C	C	C	A	T	G	T	A	C	C	C	C	T	A	A	G	G	G	G	T
R31	G	G	T	C	T	T	C	C	C	T	C	C	C	C	C	G	T	A	G	A	G	T	C	G	G	C	G	C	T	C	C	T	C	C	C	C	A	C	A	G	C	G	C	C	C	C	A	T	G	T	G	C	C	C	C	T	G	A	G	G	G	G	T
R32	G	G	T	C	T	T	C	C	C	T	C	T	C	C	C	G	T	A	G	G	G	T	C	G	G	C	G	C	T	C	C	T	C	T	C	C	A	C	A	G	C	G	C	C	C	C	A	T	G	T	G	C	C	C	C	T	G	A	G	G	G	G	T
R33	G	G	T	C	T	T	C	C	C	T	C	C	C	C	C	G	T	A	G	G	G	T	C	G	G	C	G	C	T	C	C	T	C	C	C	C	A	C	A	G	C	G	C	C	C	C	A	T	G	T	G	C	C	C	C	T	G	G	G	G	G	G	T
R34	G	G	T	C	T	C	C	C	C	C	C	C	C	C	C	G	T	A	G	G	G	T	C	G	G	C	G	C	T	C	C	C	C	T	C	C	A	C	A	G	C	G	C	C	C	C	A	T	G	T	G	C	C	C	C	T	G	A	G	G	G	G	T

R35	G G T C T T C C C T C T C C C G T A G G G T C G G C G C T C C C C T C C A C A G C G C C C C A T G T G C C C C T G A G G G G T
R36	G G T C T C C C C C T C C C C G T A G G G T C G G C G C T C C C C C C C A C A G C G C C C C A T G T G C C C C T G A G G G G T
R37	G G T C T C C C C C C C C C C G T A G G G T C G G C G C T C C C C C C C A C A G C G C C C G A T G T G C C C C T G A G G G G T
R38	G G T C T T C C C T C C C C C G T A G G G T C G A C G C T C C T C C C C A C A G C G C C C C A T G T G C C C C T G A G G G G T
R39	G G T C T T C C C C C C C C C G T A G G G T C G G T G C T C C C C C C C A C A G C G C C C C A T A T G T C C C T G A G G G G T
R40	G G T C T T C C C T C C C C C G T A G G G T C G G C G C T C C C C T C C A C A G C G C C C C A T G T G C C C C T G A G G G G T
R41	G G T C T C C C C C C C C C C G T A G G G T T G G C G T T C C C C T C C A C A A C G C C C C A T G T G C C C C T G A G G G G T
R42	G G T C T C C C C C C C C C C G T A G G G T C G G C G C T C C C C C C C A T A G C G C C C C A T G T G C C C C T G A G G G G T
R43	G G T C T C C C C C C C C C C G T A G G G T T G G C G T T C C C C T C C A C A G C G C C C C A T G T G C C C C T G A G G G G T
R44	G G T C T T C C C T C C C C C G T A G G G T C G G C G C T C C T C C C C A C A A C G C C C C A T G T G C C C C T G A G G G G T
R45	G G T C T C C T C C C C C C C G T A G G G T C G G C G C T C C C C C C C A C A G C G C C C C A T G T G C C C C T G A G G G G T
R46	G G T C T T C C C T C C C C C G T A C G G T C G G C G C T C C T C C C C A C A G C G C C C C A T G T G C C C C T G A G G G G T
R47	G G T C T T C C C C C C C C C G T A G G C T C G G C G C T C C C C C C C A C A A C G C C C C A T G T G C C C C T G A G A G G T
R48	G G C C T C C C C C C C C C C G T A G G G T C G G C G C T C C C C C C C A C A G C G C C C C A T G T G C C C C T G A G G T G T
R49	G G T C T C C C C C C C C C C G T A G G G T C G G C G T T C T C C C C T A C A G C G C C C C A T G T G C C C C T G A G G G G T
R50	G G T C T T C C C T C C C C C G T A G G G T C G G C G T T C T C C C C T A C A G C G C C C C A T G T G C C C C T G A G G G G T
R51	G A T T T T C C C T C C C C C G T A G G G T C G G C G C T C C T C T C C A C A G C G C C C C A T G T G C C C C T G A G G G G T
R52	G G T C T T C C C T C C C T C G T A G G G T C G G C G C T C C T C C C C A C A A C G C C C C A T G T G C C T C T G A G G G G T
R53	G G T C T T C C C T C C C C C G T A G G G T C G G C G C T C C T C C C C A C A G C A C C C C A T G T G C C C C T G A G G G A T
R54	G G T C T T C C C T C C C C C G T A G G G T C G G C G C T C C T C C C C A C A G C G C C C G A T G T G C C C C T G A G G G G T
R55	A G T T T C C C C C C C C C C G T A G G G T C G G C G C T C C C C T C C A C A G C G C C C C A T G T G C C C C T G A G G G G T
R56	G G T T T C C C C C C C C C C G T A G G G T C G G C G C T C C C C T C C A C A G C G C C C C A T G T G C C C C T G A G G G G T
R57	G G T T T C C C C C C C C C C G T A G G G T C G G C G C T C C C C T C C A C A G C G C C C G A T G T G C C C C T G A G G G G T
R58	A G T T T T C C C C C C C T C G T A G G G T C G G C G C T C C C C T C C A C A G C G C C C C A T G T G C C C C T G A G G G G T
R59	G G T C T C C C C C C C T C C G T A G G G T C G G C G C T C C C C C C C A C A G C G C C C G A T G T G C C C C T G A G G G G T
R60	G G T T T C C C C C C C C C C G T A G G C T C G G C G C T C C C C T C C A C A G C G C C C G A T G T G C C C C T G A G G G G T
R61	G G T C T T C C C T C C C C C G T A G G G T C G G C G C T C A T C C T C A C A G C G C C C C A T G T G C C C C T G A G G G G T
R62	G G T C T C C C C C C C C C C G T A G G G T C G G C G C T C T C C C C C A C A G C G C C C C A T G T G C C C C T G A G G G G T
R63	G G T C T T C C C T C C C C C G T A G G G T C G G C G C T C C T C C C C A C A G C G C C C C A T G T G C C C C T G A G A G G T
R64	G G T C T C C C C C C C C C C G T A G G G T C G G C G C T C C C C C C C A C A G C G C C C C A T G T G C C C C T G A A G G G T
R65	G G T C T T C C C T C C C C C G T A G G G T C G G C G C T C C T C C C C A C G G C G C C C C A T G T G C C C C T G A G G G G T
R66	G G T C C T T T C T A C C C C A C G G G G C C G G C G C T G C C T C C C A C G G T G T C C C C C G C G C T C C A G A G G G G A
R67	G G T C T T C C C T A C C C C G T A G G G T C G G C G C T C C T C C C C A C A G C G C T C C A T G T G C C C C T G A G G G G T
R68	G G T C T T C C C T C C C C C G T A G G G T C G G C G C T C C C C T C C A C A G C G C C C C A T G T G C C C C T G A G G G G T
R69	G G T C T T C C C C C C C C C G T A G G G T C G G C G C T C C C C C C C A C A G C G C C C C A T G T G C C C C T G A G A G G T
R70	G G T C T T C C C C C C C C C G T A G G C T C G G C G C T C C C C C C C A C A G C G C C C C A T G T G C C C C T G A G A G G T
R71	G G T C T C C C C C C C C C C G T A G G G T C G G C G T A C C C C C C C G C A G C G C C C C A T G T G C C C C T G A G G G G T
R72	G G T T T C C C C C C C C C C G T A G G G T C G G C A C T C C T C T C C A C A G C G T C C C A T G T G C C C T T G A G G G G T
R73	G G T C T T C C C T C C C C C G T A G G G T C G G C G C T C C T T C C C A C A G C G C C C C A T G T G C C C C T G A G G G G T

R74	G G T C T C C C C C C C C C C G T A G G G T C G G C G T T C C T C C C C A C A G C G C C C C A T G T G C C C C T G A G G G G T
