# Supplementary material for: Molecular data and ecological niche modeling reveal population dynamics of widespread shrub Forsythia suspensa (Oleaceae) in China’s warm-temperate zone in response to climate change during the Pleistocene
Source: BMC Evol Biol. 2014 May 30;14:114. doi: 10.1186/1471-2148-14-114 (PMC4052925; doi:10.1186/1471-2148-14-114)
Supplement: Additional file 3 — Geographic characteristics of 50 F. suspensa presence points used in this study. [file 1471-2148-14-114-S3.pdf]

Additional file 3 Geographic characteristics of 50 *F. suspensa* presence points used in this study.

Locations	latitude	longitude
Jigong Mt., Henan	31.83	114.08
Tongbai Mt., Henan	32.38	112.83
Longyuwan, Henan	33.70	111.75
Laojieling, Henan	33.75	111.33
Song Mt., Henan	34.47	113.08
Jiulian Mt., Henan	35.58	113.58
Hua Mt., Shaanxi	35.55	110.10
Laojun Mt., Shaanxi	34.33	110.25
Baota Mt., Shaanxi	36.58	109.48
Lingkong Mt., Shanxi	36.60	112.08
Wulaofeng, Shanxi	34.83	110.58
Wutai Mt., Shanxi	39.00	113.58
Tianlong Mt., Shanxi	37.70	112.43
Tai Mt., Shandong	36.25	117.10
Baodugu, Shandong	35.00	117.70
Yuan Mt., Shandong	36.47	117.85
Meng Mt., Shandong	35.50	117.80
Wudang Mt., Hubei	32.40	110.00
Dahong Mt., Hubei	31.52	112.97
Wuzhi Mt., Hebei	36.50	113.65
Xining, Qinghai	36.70	101.80
Qinxian, Shanxi	36.77	112.70
Qinyuan, Shanxi	36.51	112.34
Puxian, Shanxi	36.41	111.10
Jishan, Shanxi	35.61	110.98
Yangcheng, Shanxi	35.49	112.42
Yuanqu, Shanxi	35.30	111.67
Pinglu, Shanxi	34.84	111.19
Ruicheng, Shanxi	34.70	110.69
Huaxian, Shaanxi	34.52	109.77
Meixian, Shaanxi	34.28	107.75
Lushi, Henan	34.08	111.04
Shangnan, Shaanxi	33.53	110.88
Yunxi, Hubei	33.00	110.42
Liuba, Shaanxi	33.62	106.92
Lueyang, Shaanxi	33.33	106.16
Zhen'an, Shaanxi	33.43	109.15
Fangxian, Hubei	32.07	110.75
Shennongjia, Hubei	31.74	110.68
Xingshan, Hubei	31.35	110.74
Chengkou, Chongqing	31.95	108.66
Wushan, Chongqing	31.08	109.88
Wufeng, Hubei	31.20	110.67

Jinzhai, Auhui	31.74	115.93
Songxian, Henan	34.15	112.08
Badong, Hubei	31.05	110.32
Dechang, Sichuan	27.41	102.17
Kunming, Yunan	24.88	102.83
Shengyang, Liaoning	41.81	123.42
Wanning, Hainan	18.80	110.39
